# Analgesic effect of iliopsoas plane block for hip fracture

**DOI:** 10.1186/s13741-022-00254-3

**Published:** 2022-04-14

**Authors:** Chun-Guang Wang, Yang Yang, Ming-Yu Yang, Xiu-Li Wang, Yan-Ling Ding

**Affiliations:** 1 Department of Anesthesiology, The First Central Hospital of Baoding, Northern Great wall Street 320#, Baoding, 071000 Hebei China; 2 Department of Anesthesiology, The Third Hospital of Hebei Medical University, Shijiazhuang, 050051 China

**Keywords:** Nerve block, Iliopsoas plane block, Hip fracture, Hip surgery, Analgesia

## Abstract

**Background:**

Hip fracture and surgery are associated with moderate to severe pain, which hampers early mobilization and extends the hospital stay. Femoral nerve block and fascia iliaca compartment block could provide effective postoperative pain relief. Unfortunately, they could weaken the strength of the quadriceps muscle and increase the risk of falls. Iliopsoas plane block (IPB) is a novel motor-sparing regional technique, which targets the sensory branches of the hip joint originating from the femoral nerve. However, the analgesic effect of IPB has not been confirmed yet.

**Case presentation:**

In the present case series, IPB and lateral femoral cutaneous nerve block were implemented under the guidance of ultrasound for eight patients with hip fractures. The median (IQR) visual analog scale (VAS) score (0–10; 0: no pain, 10: worst pain) decreased from 1.5 (0.25–2) before IPB to 0 (0–0) 0.5h after IPB at rest. The median (IQR) VAS score decreased from 8 (7–8) before IPB to 2 (1–2) 0.5h after IPB during flexion of hip 30°. Pain score was no more than one at rest and three during flexion of the hip 30° within 48h after surgery. Furthermore, the MMT grades of quadriceps strength were no less than four after IPB.

**Conclusions:**

Our case series firstly highlights that IPB might be an effective analgesic technique for hip fracture and surgery, while retaining motor function.

## Background

It is recognized that hip fracture and surgery are associated with moderate to severe pain, which hampers early mobilization and extends the hospital stay. Peripheral nerve block technologies, such as femoral nerve block (FNB), fascia iliaca compartment block (FICB), and 3-in-1 femoral nerve block, have been used for analgesia perioperative analgesia in patients undergoing hip surgery for a long time (Li et al. 2019; Nie et al. 2015; Fournier et al. 1998). These analgesic techniques could provide effective postoperative pain relief and minimize the consumption of opioids. However, all of them could weaken the strength of the quadriceps muscle and increase the risk of falls. The pericapsular nerve group (PENG) block was successfully used for analgesia in patients with hip fracture and surgery, which was proved beneficial to early postoperative mobilization (Girón-Arango et al. 2018; Pascarella et al. 2021). However, quadriceps motor block after PENG block was reported by some recent researchers (Lin et al. 2021; Aliste et al. 2021; Yu et al. 2019).

Iliopsoas plane block (IPB), a novel motor-sparing technique described by Nielsen et al., targets selectively the sensory branches of the hip joint originating from the femoral nerve and accessory obturator nerve (Nielsen et al. 2018). Recently, a volunteer study indicated that IPB did not weaken the strength of the quadriceps muscle (Nielsen et al. 2020). However, the analgesic effect of IPB has never been confirmed. Herein, we share our experiences on the analgesic effect of IPB in eight patients with hip fractures.

## Case presentation

In this case series, eight patients (one man and seven women) with femoral neck fracture were scheduled for surgery, including an internal screw fixation, three total hip arthroplasties, and four hip hemiarthroplasties. The demographics of patients were shown in Table 1. Fasting was required routinely before operation. Intravenous access was opened, and electrocardiogram, noninvasive blood pressure, and pulse oxygen saturation were monitored routinely in the theater. All nerve block procedures were performed by the same senior anesthesiologist before anesthesia induction, whereas follow-ups were accomplished by junior anesthesiologists.

**Table 1:** Patient demographics, VAS score, MMT grades, and opioid consumption

Case	Age (year)	ASA status	BMI	Type of surgery	VAS at rest preoperative, at 0.5h after the block, in PACU and at 2, 4, 6, 24, and 48h after surgery	VAS during flexion of hip 30° preoperative, at 0.5h after the block, in PACU and 2, 4, 6, 24, and 48h after surgery	MMT grades at 0.5h after block, in PACU and 2, 4, 6, 24, and 48h after surgery	Opioid consumption 48h after surgery (morphine equivalent, mg)
1	17	I	19.0	Internal fixation	0, 0, 0, 0, 0, 0, 0, 0	0, 0, 0, 1, 1, 1, 0, 0	5, 5, 5, 5, 5, 5, 5	0
2	77	II	17.9	Hip hemiarthroplasty	1, 0, 0, 1, 1, 2, 0, 0	8, 2, 0, 2, 2, 3, 1, 1	4, 4, 4, 4, 4, 5, 5	3
3	58	II	22.2	Hip hemiarthroplasty	2, 0, 0, 1, 1, 1, 0, 0	8, 3, 1, 2, 2, 2, 1, 1	4, 4, 5, 5, 5, 5, 5	5
4	63	II	21.9	Total hip arthroplasty	1, 0, 0, 0, 0, 0, 0, 0	8, 1, 0, 0, 0, 0, 0, 0	4, 4, 4, 4, 4, 4, 5	2
5	83	II	22	Hip hemiarthroplasty	0, 0, 0, 1, 1, 1, 1, 0	7, 2, 0, 2, 2, 2, 2, 1	4, 4, 4, 4, 4, 4, 4	4
6	77	III	29.3	Hip hemiarthroplasty	2, 0, 0, 0, 0, 0, 0, 0	8, 1, 0, 0, 0, 0, 1, 1	4, 4, 5, 5, 5, 5, 5	6
7	69	II	32.3	Total hip arthroplasty	2, 0, 0, 0, 0, 0, 0, 0	8, 2, 2, 1, 1, 1, 1, 1	4, 4, 4, 5, 5, 5, 5	0
8	70	II	21.5	Total hip arthroplasty	4, 1, 2, 0, 0, 0, 0, 0	7, 2, 3, 2, 2, 2, 1, 1	4, 4, 5, 5, 5, 5, 5	0

*M* Male, *F* Female, ASA American Society of Anesthesiologists, *VAS* Visual analog scale, *MMT* Manual muscle testing, *PACU* Post-anesthesia care unit

**Fig. 1:**
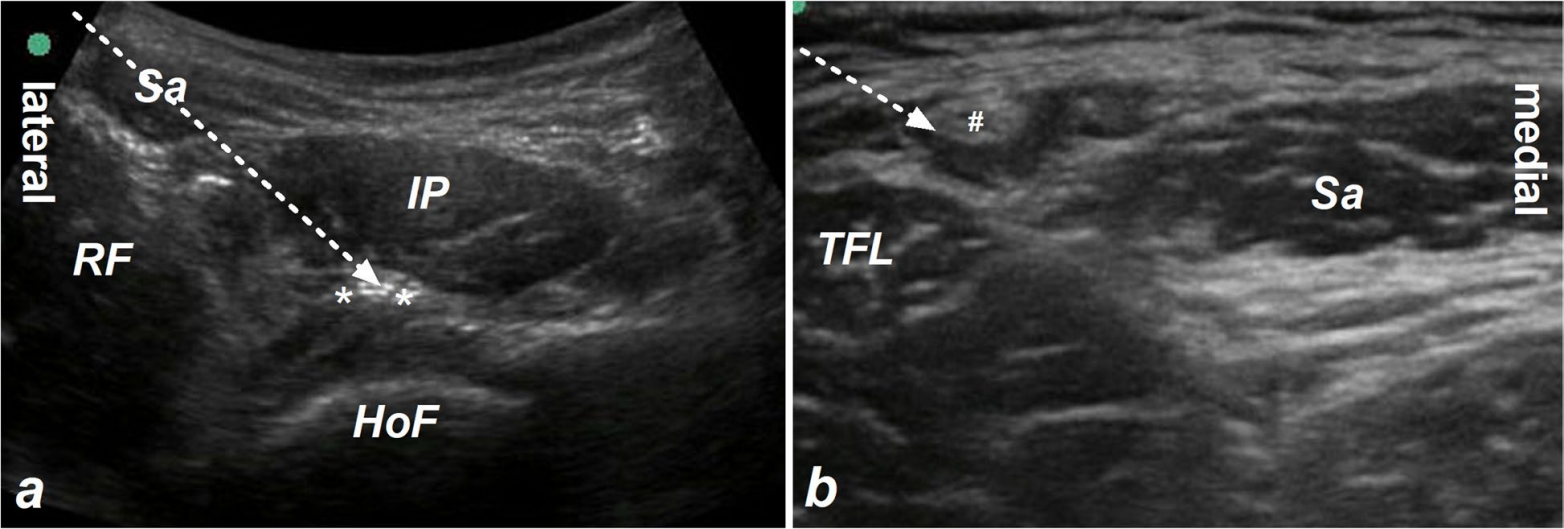
Iliopsoas plane block (**a**) and lateral femoral cutaneous nerve block (**b**). Sa indicates the sartorius muscle, RF indicates the rectus femoris muscle, IP indicates the iliopsoas muscles, HoF indicates the head of the femur, white asterisk indicates the iliofemoral ligament, TFL indicates the tensor facia latae muscle, white # indicates the lateral femoral cutaneous nerve, and white arrow indicates the needle trajectory of nerve block

Prior to general anesthesia, IPB was implemented under the guidance of ultrasound as reported by Nielsen et al. (2020). In order to evaluate the analgesic effect after IPB, any sedative drug was not given. With a supine position, a low-frequency ultrasound probe was placed distal to the anterior superior iliac spine in the transverse plane. Then, the probe was gyrated in an anticlockwise direction about 30° and slid along the inguinal ligament until the head of the femur entered the acetabular rim. After a local infiltration of 1% lidocaine, a needle was penetrated through the sartorius and iliopsoas muscle and reached into the iliopsoas plane between the iliopsoas muscle and the iliofemoral ligament (Fig. 1a). After the position of the needle tip has been confirmed, 10 ml of 0.5% ropivacaine containing 5 mg dexamethasone was injected. With 5 ml of 0.5% ropivacaine containing 2.5 mg of dexamethasone, the ultrasound-guided lateral femoral cutaneous nerve block was performed as reported by Vilhelmsen et al. (Fig. 1b) (Vilhelmsen et al. 2019).

Anesthesia induction was performed after confirming the effect of the nerve block. Propofol, remifentanil, sevoflurane, and cisatracurium were used for anesthesia induction and maintenance. Ventilation by laryngeal mask, end-tidal carbon dioxide was maintained at 35 to 40mmHg. During the operation, the bispectral index was maintained at 45 to 55, and the fluctuation of mean artery pressure and heart rate was not more than ±10% of the baseline value.

**Fig. 2:**
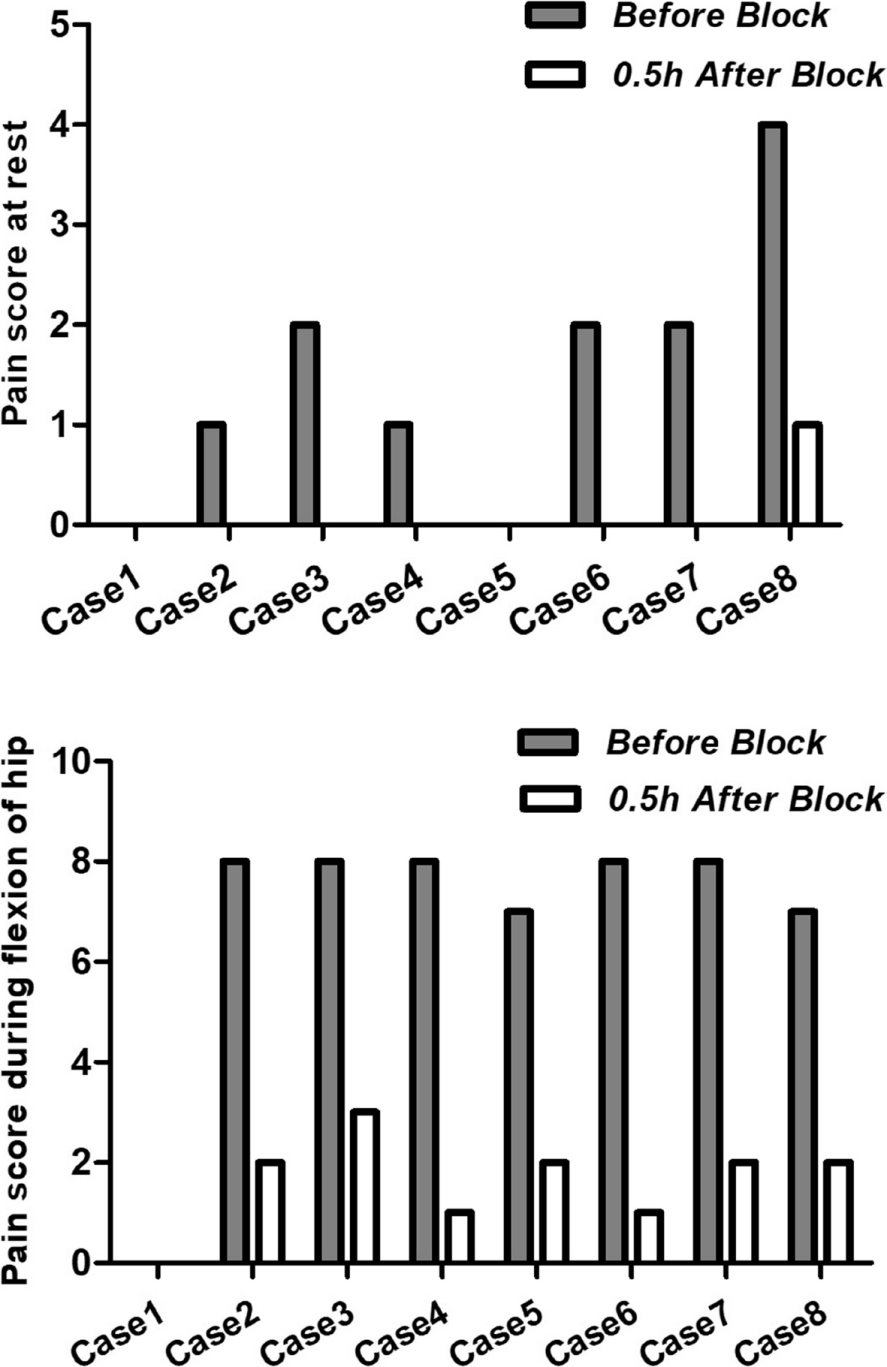
Pain score before and 0.5h after the block at rest and during flexion of the hip. The pain score at rest for cases 1 and 5 were 0 before and after the block. The pain score at rest for cases 2, 3, 4, 6, and 7 were 0 after the block. The pain score during flexion of the hip for case 1 before and after the block was 0

The operations took 60–130 min. After the operation, flurbiprofen 50 mg was administrated by intravenous injection for 3 days, twice a day. Opioids (dezocine, butorphanol, and oxycodone) were used for rescue analgesia. The postoperative pain and quadriceps strength were assessed respectively by the visual analog scale (VAS) (0–10; 0: no pain, 10: worst pain) and manual muscle testing grades (MMT grades) (0–5; 0: no muscle contraction, 5: can bear full resistance) in post-anesthesia care unit (PACU), at 0.5h after block and 2, 4, 6, 24, and 48 h after surgery. The VAS score, MMT grades, and opioid consumption were shown in Table 1 and Fig. 2.

## Discussion and conclusions

In the present case series, the median (IQR) VAS score decreased from 1.5 (0.25–2) before IPB to 0 (0–0) 0.5h after IPB at rest. The median (IQR) VAS score decreased from 8 (7–8) before IPB to 2 (1–2) 0.5h after IPB during flexion of hip 30°. Moreover, the pain score was no more than one at rest and three during flexion of the hip at 30° within 48h after surgery. These results suggested IPB could improve pain effectively of hip fracture and surgery, which were consistent with our assumptions. Furthermore, the MMT grades of quadriceps strength were no less than four after IPB. All patients were able to fully participate in physiotherapy, and there were no falls happened in the hospital. The above results suggested IPB could provide good pain relief, while retaining motor function. Although a recent volunteer study indicated that IPB did not weaken the strength of the quadriceps muscle (Nielsen et al. 2020), the MMT grades were four after IPB in this case series. This divergence might be interpreted by the discrepancy of local anesthetic volumes (5 ml vs 10 ml). The two-fold increase in volume for IPB might lead to enlarge the spread of ropivacaine along the articular branches to the trunk of the femoral nerve, causing a motor block. The optimum capacity of local anesthetics for IPB needs to be explored in future research.

The innervation of the hip joint is complicated. The great majority of nociceptors are located in the anterior part of the capsule of the hip joint rather than the posterior capsule, which indicates that the anterior capsule is the main target of postoperative analgesia after hip surgery (Simons et al. 2015). According to the evidence of the neuroanatomy of the hip, the anterior capsule was innervated by the femoral nerve, obturator nerve, and accessory obturator nerve (if exist) (Birnbaum et al. 1997; Short et al. 2018). A recent study suggested that obturator nerve block could not improve pain after hip surgery, but would increase the risk of adductor paralysis (Nielsen et al. 2019). Therefore, with good reasons, we believe that the femoral nerve is the key target for postoperative analgesia after a hip surgery. However, FNB could paralyze the quadriceps muscle, delay discharge, and even increase the risk of fall (Kuchálik et al. 2017). PENG block, as a motor-sparing technique, was confirmed effectively on analgesia for patients with hip fracture and surgery (Girón-Arango et al. 2018; Pascarella et al. 2021). However, some recent research reported that PENG blocks could not seem to circumvent a motor block. Aliste et al. found that 45–50% of subjects with PENG block experienced some paresis or paralysis of knee extension (Aliste et al. 2021). The same result was revealed in the study by Lin et al. (2021). PENG block targets the higher branches of the femoral nerve proximal to the inguinal ligament, which causes a spread toward the trunk of the femoral nerve easily. On the contrary, IPB targets the lower sensory branches of the hip joint that originated from the femoral nerve (Nielsen et al. 2018). Moreover, the discrepancy of the local anesthetic capacity used for PENG block and IPB could be another explanation. The capacity of ropivacaine for IPB is significantly less than the PENG block. The four-fold increase of the capacity for PENG block may cause the extensive spread of local anesthetic along the articular branches to the trunk of the femoral nerve, resulting in quadriceps weakness (Endersby et al. 2021). More neuroanatomical studies and clinical trials are needed to be explored about the difference in analgesic effect and motor block between PENG block and IPB for hip fracture and surgery.

In conclusion, IPB may be an effective analgesic technique for hip fracture and surgery, while retaining motor function. More studies are needed to further confirm the validity of IPB and its optimum volume of local anesthetic.

## Data Availability

The datasets used and/or analyzed during the current study are available from the corresponding author on reasonable request.

## Abbreviations

FNB: Femoral nerve block
FICB: Fascia iliaca compartment block
IPB: Iliopsoas plane block
VAS: Visual analog scale
MMT: Manual muscle testing
PACU: Post-anesthesia care unit
